# The potential and effects of saline-alkali alfalfa microbiota under salt stress on the fermentation quality and microbial

**DOI:** 10.1186/s12866-021-02213-2

**Published:** 2021-05-19

**Authors:** Duo Wen Sa, Qiang Lu, Zhen Wang, Gentu Ge, Lin Sun, Yushan Jia

**Affiliations:** 1 College of Grassland Resources and Environment, Inner Mongolia Agricultural University, jys_nm@sina.com, Erdos Street, Hohhot, 010019 Inner Mongolia China; 2 Institute of Grassland Research, Chinese Academy of Agricultural Sciences, Hohhot, China; 3 Inner Mongolia Academy of Agriculture Animal and Husbandry Sciences, Hohhot, Inner Mongolia Autonomous Region China

**Keywords:** Alfalfa, Cellulase, Microorganisms, Salinized land, Silage

## Abstract

**Background:**

The objective of this study was to evaluate the chemical compositions and microbial communities of salt-tolerant alfalfa silage. Salt-tolerant alfalfa was ensiled with no additive control, and cellulase for 30 and 60 to 90 days. In this study, the dry matter (DM) content of the raw material was 29.9% DM, and the crude protein (CP) content of the alfalfa was 21.9% CP.

**Results:**

After 30 days of fermentation, the DM content with the cellulase treatment was reduced by 3.6%, and the CP content was reduced by 12.7%. After 60 days of fermentation, compared with alfalfa raw material, the DM content in the control group (CK) was reduced by 1%, the CP content was reduced by 9.5%, and the WSC (water-soluble carbohydrates) content was reduced by 22.6%. With the cellulase, the lactic acid content of the 30- and 60-day silages was 2.66% DM and 3.48% DM. The content of *Firmicutes* in salinized alfalfa raw material was less than 0.1% of the total bacterial content. Before and after ensiling, the microbes had similar composition at the phylum level, and were composed of *Firmicutes*, *Actinobacteria*, *Bacteroidetes,* and *Proteobacteria*. The abundance of *Pantoea* was dominant in fresh alfalfa. In the absence of additives, after 30 days and 60 days of silage, the dominant lactic acid bacteria species became *Lactococcus* and *Enterococcus*.

**Conclusions:**

The results showed that LAB (*Lactobacillus*, *Lactococcus*, *Enterococcus,* and *Pediococcus*) played a major role in the fermentation of saline alfalfa silage. It also can better preserve the nutrients of saline alfalfa silage. The use of cellulase enhances the reproduction of *Lactobacillus*. The fermentation time would also change the microbial community of silage fermentation.

**Supplementary Information:**

The online version contains supplementary material available at 10.1186/s12866-021-02213-2.

## Background

With the rapid development of animal husbandry around the world, the availability of forage with adequate and high protein content is in increasingly high demand [1]. It has become increasingly important to obtain more protein feed from limited land resources. Salinized soil resources were distributed in more than 100 countries around the world, and there is a global salinized land area of 955 million hm^2^ [2]. Soil salinization is an issue affecting the development of global agriculture and animal husbandry. Alfalfa [*Medicago sativa* L.] has high levels of crude protein, digestible nutrients, minerals [3]. Alfalfa could adapt to the salinized environment and can be planted in saline lands [4]. Alfalfa is not only showing high salt tolerance to saline-alkali soil but also appropriate salt stress would improve the quality of alfalfa, including amino acids, proteins, and other important nutrients [5, 6]. Alfalfa in salinization land can not only provide a large amount of high-quality protein feed for animal husbandry but also can use the rhizobium of alfalfa to improve the soil quality of saline-alkali land [7]. It grows well in neutral salinized or lightly salinized soils, and greatly increases the utilization rate of salinized land [8]. Therefore, salt-tolerant alfalfa may be a potential feed source.

Forage processing could play a positive role in promoting intake and digestion [9]. Ensiling is a method of preserving fresh forage that involves anaerobic fermentation of lactic acid bacteria (LAB) to preserve nutrients effectively [10]. During the anaerobic fermentation, water-soluble carbohydrates (WSC) are metabolically decomposed into lactic acid by LAB until the pH drops to about 4.5. Additives can also improve the quality of the silage [11]. Common additive types include fermentation promoter and fermentation inhibitors. Cellulase is an important fermentation promoter. It can effectively preserve the nutritional value of the forage [12]. Given the need to make full use of salinized land resources and expand the forage sources, the purpose of the study was to investigate the influence of time and additives on salt-tolerant alfalfa fermentation characteristics and the bacterial community of silage, to improve the quality of alfalfa under salinization.

## Methods

### Silage preparation

We selected the Zhongmu No. 3 (salt-tolerant) alfalfa variety. The alfalfa was harvested on July 15, 2018, in an experimental field at Inner Mongolia Agricultural University (40° 17′ N, 111° 27′ E). The fresh alfalfa was treated by air drying for 4 hours. It was then treated with cellulase or no cellulase (i.e., control treatment; CK), and ensiled for 30 days and 60 days. The ensiled alfalfa was chopped into 2–3 cm. The material was placed in polyethylene plastic bags (20 × 30 cm), each bag containing 300 g, with three replicates per treatment. A vacuum-packaging machine was used to seal the bags. The silage samples were stored indoors.

### Analysis of microbial population and chemical composition

After 30 and 60 days, the alfalfa silage bags were opened, 10 g sample from each bag were mixed with 90 mL of sterile aqueous solution [13], and the fermentation broth fully extracted using a homogenous slap apparatus. The bacterial solution was diluted from 10^− 1^ to 10^− 5^ and used to count the number of microorganisms. The amount of LAB was calculated using MRS medium under anaerobic conditions, and the amount of *Escherichia coli* was calculated using Rose Bengal Agar under aerobic conditions.

The fermentation broth was used to determine the fermentation quality of alfalfa, using the method of Wright [14]. The content of organic acids was determined using a liquid chromatograph. The pH value of the silage was determined using a glass electrode pH meter (STARTER 100/B, OHAUS, Shanghai, China). The dry matter (DM) content was calculated after drying the tantalum sample at 65 °C for 48 h. The crude protein (CP) content was determined by the Chen’ method [15]. The neutral detergent fiber (NDF) and acid detergent fiber (ADF) contents were determined as described by Van et al. [16]. The water-soluble carbohydrate (WSC) content was determined as described by Thomas [17].

### DNA extraction and PCR amplification

Silage microbial DNA was extracted according to the EZNA® kit instructions (Omega Bio-tek, Norcross, GA, US). The concentration and purity of the DNA were determined using a NanoDrop 2000 (Thermo Scientific, Wilmington, USA). The DNA extraction quality was measured by 1% agarose gel electrophoresis 338F (5′-ACTCCTACGGGAGGCAGCAG-3′) and 806R (5′-GGACTACHVGGGTWTCTAAT-3′). Primers were used for PCR amplification of the V3-V4 variable region. The amplification procedure was: pre-denaturation at 95 °C for 3 min., 27 cycles (denaturation at 95 °C for 30 s, annealing at 55 °C for 30 s, extension at 72 °C for 30 s), extended for 10 min. at the end at 72 °C (PCR instrument: ABI GeneAmp® 9700). The amplification system with a total volume of 20 μL contained 4 μL 5*FastPfu buffer solution, 2 μL 2.5 mM dNTPs, 0.8 ul primer (5 μM), 0.4 μL FastPfu polymerase, and 10 ng DNA template [18]. The resulting PCR product was extracted from a 2% agarose gel and further purified using an AxyPrep DNA Gel Extraction Kit (Axygen Biosciences, Union City, CA, USA) and QuantiFluorTM-ST (Promega, USA) according to the manufacturers’ instructions.

Raw fastq files were demultiplexed, quality-filtered by Trimmomatic, and merged by FLASH with the following criteria: (i) the reads were truncated at any site receiving an average quality score < 20 over a 50 bp sliding window; (ii) primers were exactly matched allowing 2 nucleotide mismatching, and reads containing ambiguous bases were removed; (iii) sequences whose overlap was longer than 10 bp were merged according to their overlap.

Operational taxonomic units (OTUs) were clustered with 97% similarity cutoff using UPARSE, and chimeric sequences were identified and removed using UCHIME. The taxonomy of each 16S rRNA gene sequence was analyzed by RDP Classifier algorithm against the Silva (SSU123) 16S rRNA database using a confidence threshold of 70%.We uploaded the sequences data in the NCBI and got an accession number PRJNA560790.

### Statistical analysis

SAS 9.3 software was used to analyze the differences of datas in the article. The difference between the means was assessed by Tukey’s multiple comparison test, at a significance level of *P* < 0.05.

## Results

### Silage characteristics of fresh alfalfa

The nutrient composition of fresh alfalfa and the composition of microbial community are shown in Table 1, nutrient indicators were calculated based on the dry matter (DM) content of the raw material. The DM content was 29.9%, and the crude protein (CP) content of the alfalfa was 21.9% DM. The water-soluble carbohydrates (WSC) content was 3.27% DM. The acid detergent fiber (ADF) and neutral detergent fiber (NDF) contents were 33.9 and 37.4% DM. The amount of LAB was 4.57 CFU/g FM, higher than the amount of yeast. The quantity of LAB was sufficient to start ensiling.

**Table 1:** Chemical and microbial compositions in the pre-ensiled samples

Items	*Medicago sativa* L.
DM^a^ (%)	29.9
Crude protein (% DM)	21.9
Neutral detergent fiber (% DM)	37.4
Acid detergent fiber (% DM)	33.9
Fatty acid (% DM)	7.4
Water-soluble carbohydrates (% DM)	3.27
Lactic acid bacteria (Log CFU /g FM)^b^	4.57
Coliform bacteria (Log CFU /g FM)^b^	4.8
Yeast (Log CFU /g FM)^b^	2.24

^a^*DM* Dry matter

^b^*CFU* Colony-forming units

### Nutritional and fermentation quality of alfalfa silage

After 30 and 60 days of anaerobic fermentation, the nutritional quality and microbial community of the silage are shown in Table 2. After 30 days of fermentation, the CP and WSC contents changed significantly (*P*<0.05). Compared with raw alfalfa, the DM content with cellulase treatment was reduced by 3.6% and the CP content was reduced by 12.7%, a greater reduction than in the CK. The change law showed the same pattern in the WSC. After 60 days of fermentation, compared with raw alfalfa, the DM content in the CK was reduced by 1%, the CP content reduced by 9.5%, the WSC content reduced by 22.6%, and the DM content in the cellulase treatment reduced by 5.3%.

**Table 2:** Chemical characteristics of silage prepared with and without cellulase under ensiling for 30 and 60 days

Items	30 days		60 days	
	CK‡	Cellulase	CK	Cellulase
DM (%)	30.3a	28.8b	29.6b	28.3a
Crude protein (% DM†)	20.7a	19.1a	19.8a	18.4a
Neutral detergent fiber (% DM)	42.3b	41.9a	41.9a	39.8a
Acid detergent fiber (% DM)	38a	36.7a	39.3a	38.3a
Fatty acid (% DM)	1.87a	2.18a	2.1a	1.95a
Water-soluble carbohydrates (% DM)	27.06a	30.2a	25.3b	27.6b

†*DM* Dry matter

‡*CK* Control (no addition)

Values within the same row under the same ensiling days with different superscripts in lowercase letters differ significantly from each other at *P* < 0.05

The quality of fermentation was also crucial and is shown in Table 3. Butyric acid, which has been bad for livestock, was not detected, indicating that the silage had good palatability. Lactic acid is the main product of LAB in the process of fermentation and metabolism. With cellulase, the lactic acid content of the 30 and 60 days silages were 2.66 and 3.48%, indicating an increasing of 23.5% in the 30 days period. Its content was higher than CK. The content of acetic acid in the CK increased from 4.63% (30 days) to 4.7% (60 days), an increase of 1.4%, and the content of acetic acid in the cellulase treatment increased from 3.48% (30 days) to 4.85% (60 days), an increase of 28.2%. With the extension of the fermentation time, the pH value decreased and the NH_3_-N content increased in the both CK and the cellulase treatment. In the CK, the NH_3_-N content increased from 2.38% (30 days) to 2.45% (60 days), an increase of 2.8%, while the increase with the cellulase treatment was 22.8%.

**Table 3:** CHemical fermentation characteristics of alfalfa silage prepared with and without cellulase under ensiling for 30 and 60 days

Items	30 days		60 days	
	CK‡	Cellulase	CK	Cellulase
pH	4.37a	4.27a	4.24b	4.1a
Lactic acid (% DM†)	2.39b	2.66b	2.6a	3.48a
Acetic acid (% DM)	4.63a	3.48b	4.7a	4.85a
Propionic acid (% DM)	0.01b	0.01a	0.01b	0.01a
Butyric acid (% DM)	ND§	ND	ND	ND
NH3-N (% DM)	2.38b	1.92a	2.45b	2.49a

†*DM* Dry matter

‡*CK* Control (no addition)

§Not detected

Values within the same row under the same ensiling days with different superscripts in lowercase letters differ significantly from each other at *P* < 0.05

### Bacterial diversity of salt-tolerant alfalfa silage during the fermentation process

As can be seen from Table 4, the coverage of all samples was higher than 99%. This indicates that the sequencing width was relatively comprehensive and the microbial high-throughput data were sufficient to represent the characteristics of the bacterial microbial community. After 60 days of fermentation, the number of OTUs decreased, which indicate that the LAB had become the dominant flora, inhibiting the growth and reproduction of harmful microorganisms. The number of OTUs and the Chao index differed according to additive use and ensiling duration; both fermentation time and cellulase treatment reduced the alpha diversity of microorganisms.

**Table 4:** Alpha diversity of bacterial diversity at 30 and 60 days of ensiling

Items		OTU	Shannon	Ace	Chao	Coverage
	M^a^	132	2.16	112.7	111.35	0.99
30 days	CK^b^	90	1.29	105.79	84.29	0.99
	Cellulase	131	1.71	102.95	98.11	0.99
60 days	CK	128	1.83	102.38	102.7	0.99
	Cellulase	110	1.48	103.57	93.32	0.99

^a^*M* Pre-ensiled alfalfa

^b^*CK* Control (no addition)

**Fig. 1:**
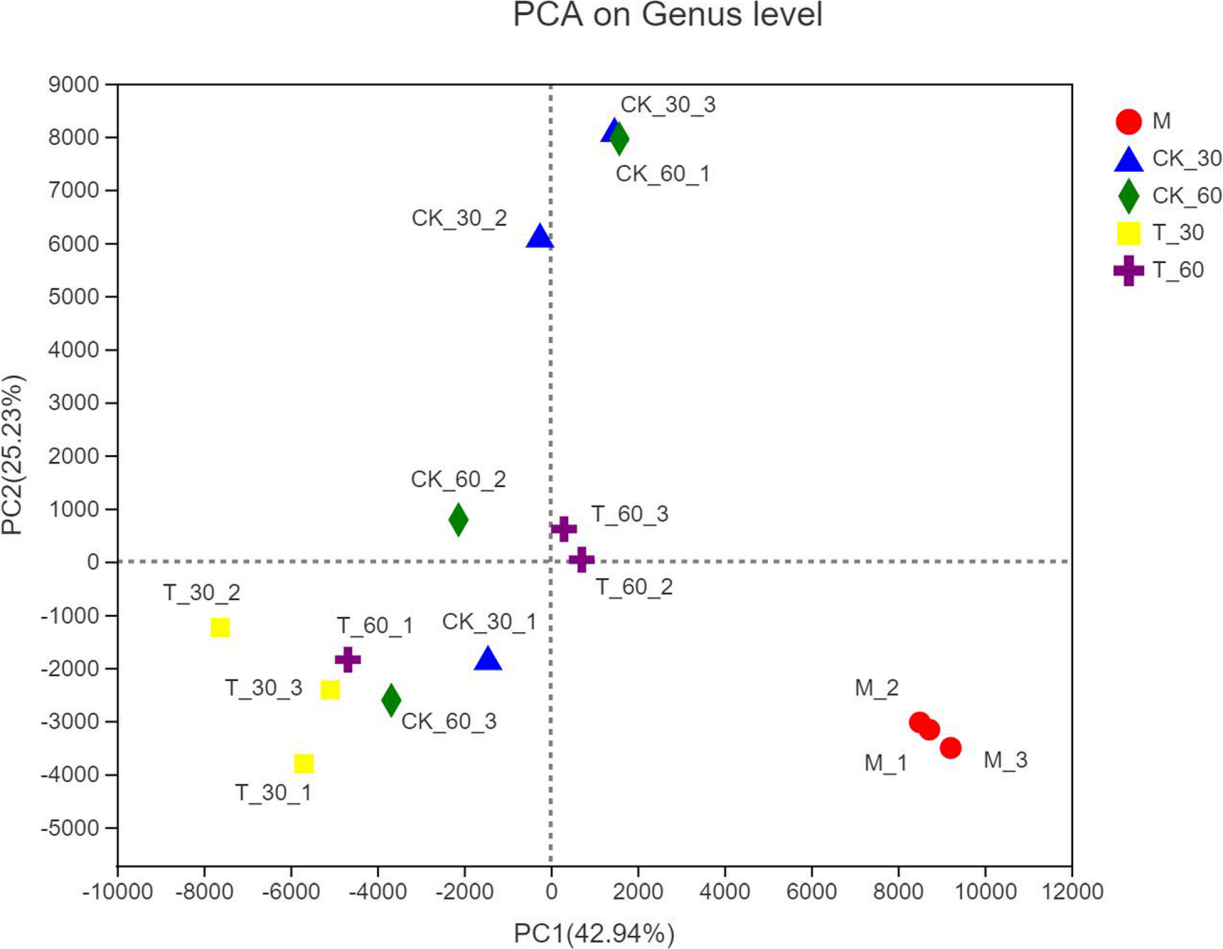
Principal component analysis (PCA) of samples. PC1, principal component 1; PC2, principal component 2; CK, control (no addition); 30, ensiled for 30 days; 60, ensiled for 60 days; T_30, samples added for treatment, the same as other groups; CK_60, control ensiled for 60 days, the same as other groups

Principal component analysis (PCA) analyzed the similarities and differences between bacterial community with different treatments and different fermentation times. As shown in Fig. 1, the contribution of PCA 1 to the interpretation of total variance was 42.94%, while PCA 2 explained 25.23% of the total variance. Overall, there was a large difference between raw alfalfa and silage samples. The microbial flora of fresh alfalfa were quite different from the silage. The areas of CK_30 and CK_60 had a large overlap and the difference was small, while the difference between T_30 and T_60 was large.

**Fig. 2:**
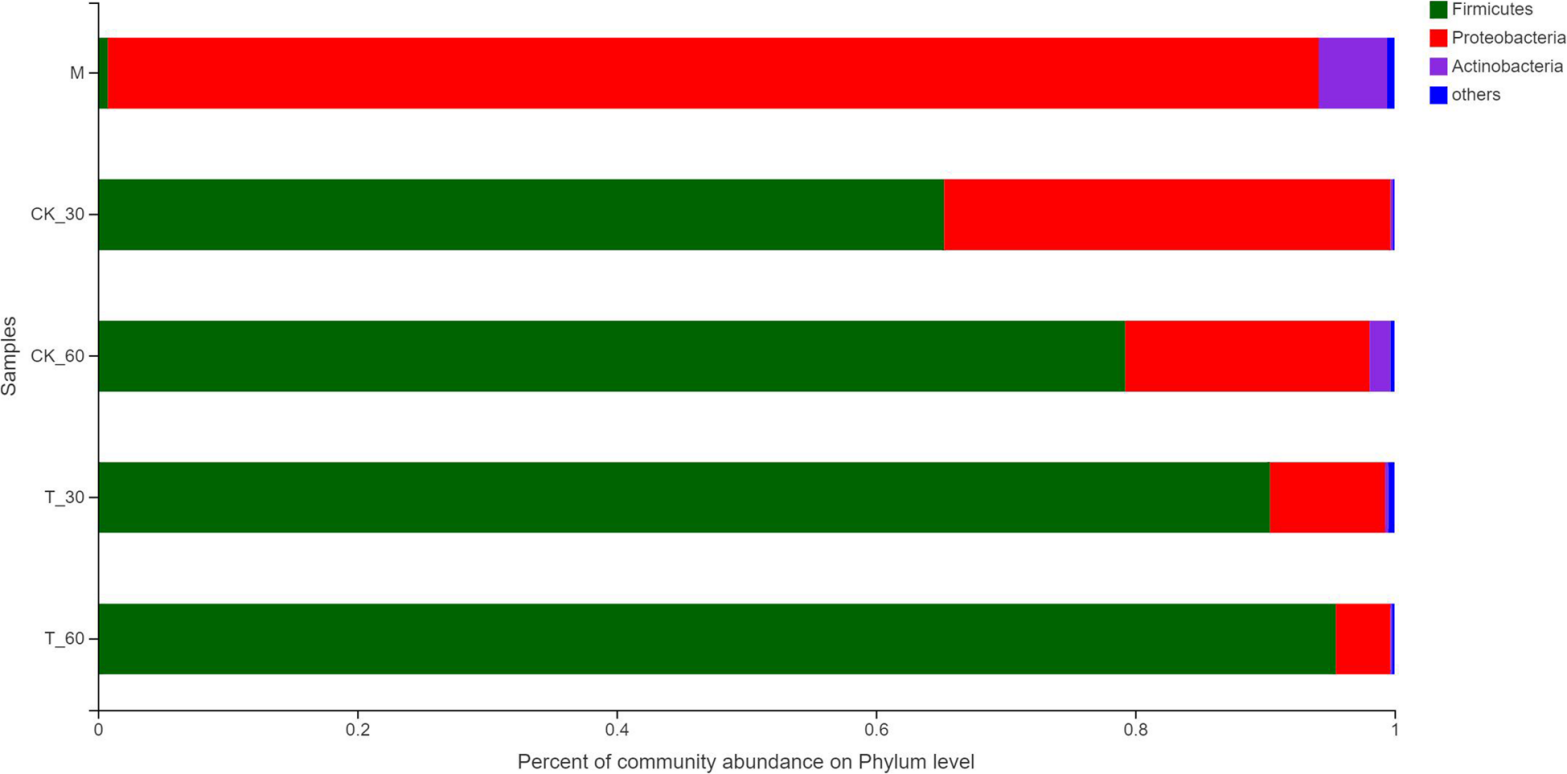
Relative abundance of bacteria at the phylum level. M, pre-ensiled sample; CK, control (no addition); T, with addition of cellulase; 30, ensiled for 30 days; 60, ensiled for 60 days; the same as other groups; 1, 2, 3, triplicate per treatment

The microbial community composition of alfalfa silage was represented by mainly phylum (Fig. 2). The content of *Firmicutes* in raw alfalfa was less than 0.1%, and *Proteobacteria* was the most predominant phylum. In the CK and cellulase treatment, with the extension of the fermentation time, the abundance of *Firmicutes* gradually increased, but the abundance of *Proteobacteria* was decreased.

Among the microbes, the abundance of *Proteobacteria* was as high as 92.17% in the pre-ensiled materials. However, after 30 days and 60 days of silage fermentation, the abundance of *Proteobacteria* decreased from 92.17% to 1.75–36.13%. The abundance of *Firmicutes* increased from 0.99% (M) to between 63.6 and 93.9%. As the duration increased, the abundance of *Firmicutes* increased in both the control silage group and the cellulase treatment silage (T) by 3 to 13%. In different experimental addition treatments, the increasing in *Firmicutes* content of silage with added cellulase was greater, and the *Firmicutes* (60 days) content was relatively higher.

**Fig. 3:**
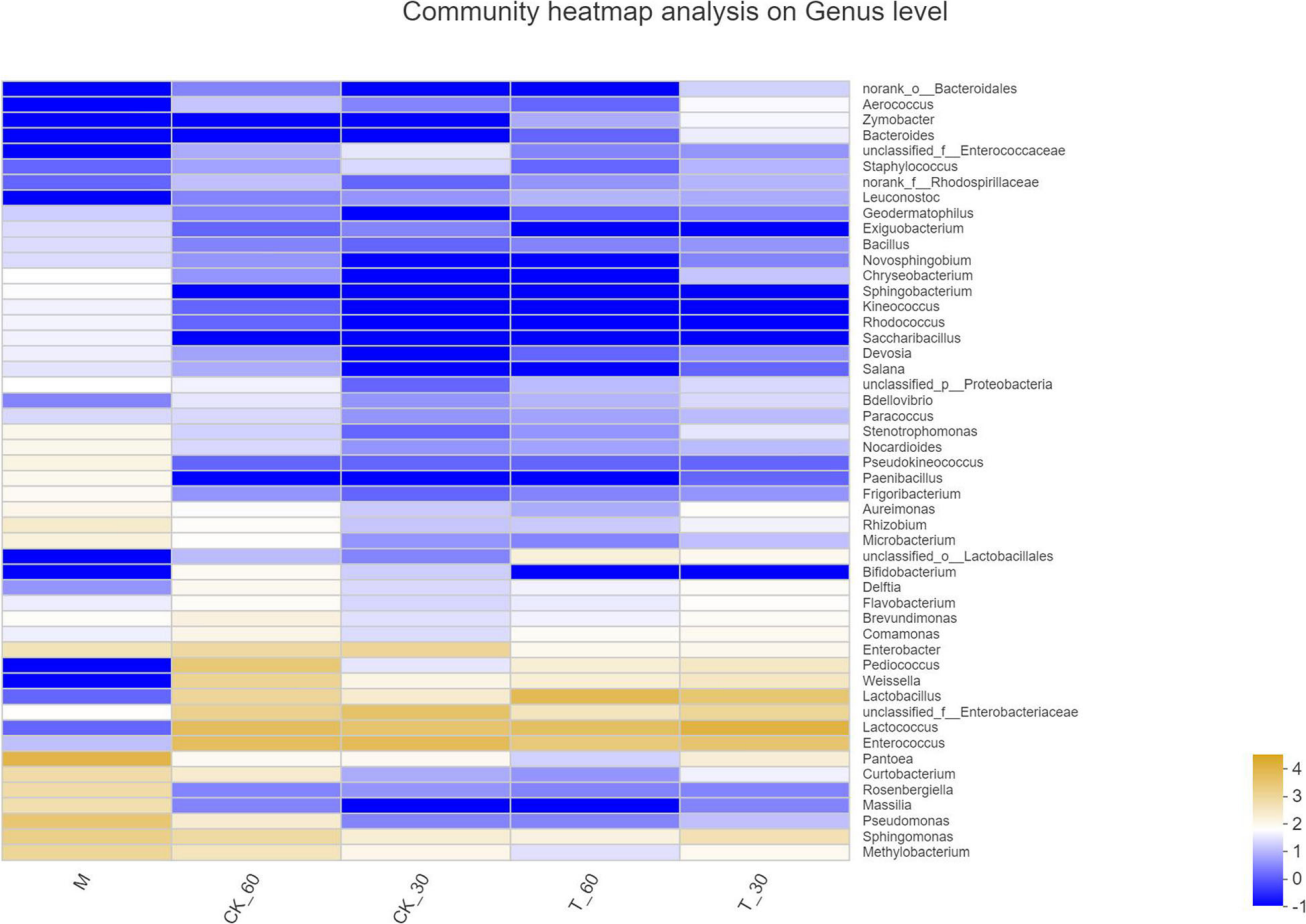
Heat map of bacteria at the genus level. M, Pre-ensiled sample; CK, control (no addition); T, with addition of cellulase; 30, ensiled for 30 days; 60, ensiled for 60 days; the same as other groups; 1, 2, 3, triplicate per treatment

Changes in the bacterial community composition during the fermentation process at the genus level are shown in Fig. 3. As illustrated, the abundance of *Pantoea* was dominant in the fresh alfalfa, and the advantage of LAB were *Lactococcus* and *Enterococcus*. It may also be that the LAB suitable for growth on the alfalfa of saline were *Lactococcus* and *Enterococcus*. In the absence of additives, after 30 days and 60 days of silage, the dominant LAB became *Lactococcus* and *Enterococcus*. The dominant LAB did not change greatly. Under the influence of the fermentation promoter, there was a slight difference from the CK, as the dominant LAB increased *Lactobacillus*.

**Fig. 4:**
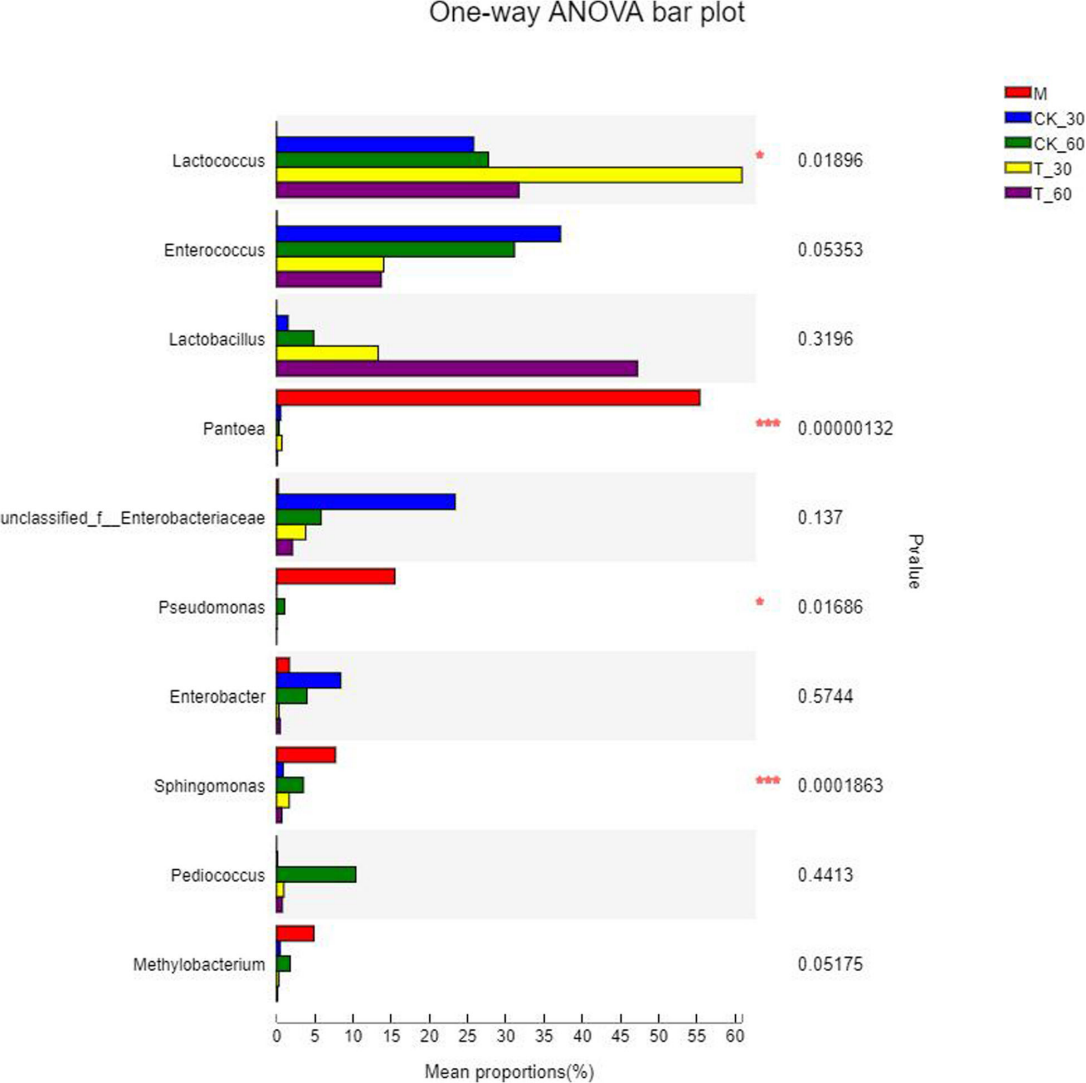
Comparison of microbial variations using the one-way ANOVA for alfalfa silage

To further reveal the succession of bacterial communities of alfalfa silage, bacterial communities at genus level are shown in Fig. 4. *Pantoea* was the dominant genus in the CK (55.4%). *Lactococcus* was 25.79 and 27.72% in CK_30 and CK_60. But *Lactococcus* became 60.91% in T_30, 47.95% higher than T_60 (31.7%). *Enterococcus* was in the treatment (14.02, 13.7%) lower than CK (31.12, 37.15%). With the extension of fermentation time, the content of *Lactobacillus* in CK_60 (4.84%) and T_60 (47.22%) was more than CK_30 (1.48%) and T_30 (13.3%).

The inductive organization of silage sample microorganisms, through each OTU corresponding gene ID, from the KEGG database to the description of each pathway and its functional information, thereby obtaining functional abundance, is in Table 5. The abundance of amino acid metabolism was 1734546ab, which was significantly different from metabolism of other acids (*P* < 0.05). The microbial activities of alfalfa silage were mainly in amino acid metabolism, glycan biosynthesis and metabolism, enzyme families, carbohydrate metabolism, metabolism of other amino acids, xenobiotics biodegradation, and metabolism. With fermentation, the abundance of carbohydrate metabolism in CK_30 (2595879a) and T_30 (1366396b) dropped to CK_60 (1708199ab) and T_60 (878991b). Enzyme families, biosynthesis of other secondary metabolites, amino acid metabolism, cellular processes and signaling, glycan biosynthesis and metabolism, carbohydrate metabolism, metabolism of other amino acids, xenobiotics biodegradation, and metabolism showed the same regular pattern.

**Table 5:** Statistics of abundance of alfalfa silage microorganism KEGG pathway

Items	M†	CK‡_30	CK_60	T§_30	T_60
Biosynthesis of other secondary metabolites	144487ab	151372a	106654abc	92316bc	53808c
Amino acid metabolism	1734546ab	2025808a	1331435abc	1096366bc	648071c
Cellular processes and signaling	880958ab	1337523a	713491bc	496637bc	272306c
Glycan biosynthesis and metabolism	429512ab	574628a	329849bc	254812bc	150012c
Enzyme families	366165ab	513575a	311147bc	237307bc	147550c
Carbohydrate metabolism	1827729ab	2595879a	1708199ab	1366396b	878991b
Metabolism of other amino acids	334661ab	422054a	274368abc	222434bc	135539c
Signaling molecules and interaction	22710a	19672a	28117a	31392a	30907a
Xenobiotics biodegradation and metabolism	501238a	522642a	384554ab	345977ab	204920b
Environmental adaptation	25369a	26743a	18606ab	13691b	8913b

†*M* Pre-ensiled alfalfa

‡*CK* Control (no addition)

§*T* Treatment (with addition)

Values within the same row under the same ensiling days with different superscripts in lowercase letters differ significantly from each other at *P* < 0.05

## Discussion

Ensiling is a process in which saccharide is converted into organic acid by LAB fermentation in an anaerobic environment to reduce the pH and inhibit the growth and reproduction of harmful microorganisms. This prevents the loss of nutritional value in feed. Good silage preservation requires a LAB count of > 10^5^ CFU/g FM. In this study, the raw material had a low LAB content (< 5.00 log CFU/g FM), less than the number of beneficial microorganisms to support the success of the experiment. The data show that *E. coli* content was high, which indicates that alfalfa silage requires additives to ensure complete fermentation.

Adequate WSC is a key factor in forage silage, which provides sufficient nutrient substrate for LAB reproduction. When the content of WSC reaches 60–80%, fermentation can be carried out normally [19]. In this study, the WSC content of the alfalfa was 3.27% DM, which was lower than the WSC content of silage maize [1]. It was possible that salt stress in soil inhibits the accumulation of WSC in fresh alfalfa [6]. Therefore, the current WSC content is sufficient to ensure good preservation of the alfalfa with additives. This research showed a relatively high CP content, which may be due to salt stress promoting protein accumulation in plants.

The fermentation time has an important influence on silage fermentation quality and microorganisms [20]. After 30 days of ensiling, the content of CP and WSC decreased, due to LAB fermentation. This was similar to the results of Maharlooei [21]. It is known that cellulase has a degrading effect on macromolecular carbohydrates such as cellulose, hemicellulose, and lignin in the crude fiber of the stem, which degrade into small molecules of monosaccharides or polysaccharides, thereby rapidly enhancing the fermentation activity of LAB. Cellulase has a significant effect on lactic acid content, pH, and NH_3_-N. As the efermentation time increased, the microorganisms of LAB became increasingly active. Key nutrients were gradually being consumed, but the content of some, such as propionic acid and acetic acid, did not differ. It is also apparent that there is no spoilage in the fermentation process, and the rapid propagation of LAB, low pH, and the anaerobic environment inhibited the growth of mold. These findings suggested that salt-tolerant alfalfa had better nutritional quality after cellulase was added. Comparing the effects of additives and fermentation time, the fermentation time had a great effect on loss of nutritional quality. However, the changes in the treatments with the same fermentation time were consistent. From the nutritional point of view, salt-tolerant alfalfa’s overall quality is better. This indicates that salt-tolerant alfalfa could be used as forage for animals after fermentation.

High-throughput determinations were performed of variable regions 3 and 4 of 16 s rDNA to calculate and evaluate bacterial diversity after ensiling. Before and after the fermentation, the microbes had similar composition at the phylum level, which were composed of *Firmicutes*, *Actinobacteria*, *Bacteroidetes*, and *Proteobacteria*. The only difference was the changes in abundance. The changes in the relative abundance of different genus of bacteria reflected differences in the response to the treatments. The main microorganisms in the silage of the salt-tolerant alfalfa were still the *Firmicutes*, but the main genus had changed from *Enterococcus* (30 days) to *Lactococcus* (60 days). After the addition of cellulase, the dominant genus shifted from *Lactococcus* (30 days) to *Lactobacillus* (60 days) [22]. Jacxsens et al. revealed that *Pantoea* would be metabolized to produce acetic acid, propionic acid, and succinic acid [23]. *Enterobacteriaceae* is also capable of producing carbohydrate metabolism under anaerobic conditions. This is similar to the results of this study. The relative abundance of *Enterobacteriaceae*, *Lactococcus* and *Pantoea* were higher in the silage under salt stress (Fig. 3), which may explain the increase in organic acids.

Lactic acid is the main factor leading to the pH drop in the silage, and pH is also an important indicator of whether anaerobic fermentation was complete. McDonald also showed that pH is an important indicator of the degree of fermentation and the quality of silage [22]. In the experiment, the organic acid content at 60 days was significantly higher than at 30 days, and the nutrient quality of alfalfa silage under salt stress was better. However, after 30 and 60 days fermentation, the cellulase-added silage had a higher WSC content, but its CP content was lower than in the CK. WSC is decomposed into lactic acid water by glycolysis (EMP) or the hexose phosphate (HMP) pathway. This may be because the addition of cellulase leads to a dramatic increase in the number of lactic acid bacteria and expands the effect of anaerobic fermentation [24]. On the other hand, alfalfa has been identified as having high antibacterial activity [25], which may inhibit the growth of microbes. In the current, as fermentation time prolonged, the lactic acid content increased and the pH value decreased significantly. This may be because *Lactobacilli* can metabolize lactic acid in the absence of carbohydrate [26].

High-throughput sequencing can provide a wealth datas for exploring taxonomic classifications and activities of silage microbial community [27]. In this study, alpha diversity values indicate that the diversity of bacterial communities after silage was higher, which was consistent with the results of Li [28]. St-Pierre found that *Mencius*, *Bacteroides*, *Chloroflexi*, and *Proteobacteria* were dominant phyla that played an important role in hydrolysis and acid production [29]. *Firmicutes* is the main phylum was the most in grass silage [30]. *Proteobacteria* are the most abundant bacteria in fresh alfalfa, and the content is above 90%. Bao also found that *Proteobacteria* is the main phylum of fresh alfalfa [31]. In our study, the main genera after ensiling (e.g., *Lactobacillus*, *Enterococcus*, *Lactococcus*, and *Weissella*) were also significantly inhibited during the fermentation process. The growth of spoilage bacteria such as *Proteobacteria* is similar to the findings of Yanbing in experiments on corn and ryegrass [32].

LAB is an important member of the bacterial community in silage, with major effects on silage quality. *Lactobacillus*, *Lactococcus*, and *Enterococcus* played key roles in the anaerobic fermentation of salt-tolerant alfalfa. Even *Enterobacteriaceae* and *Pantoea* played a role in, and they also consume nutrients for growth and reproduction. Their relative abundance was lower than the LAB, which also showed that the silage was well fermented. Under the influence of cellulase in silage, *Lactobacillus* rapidly multiplies and became another dominant bacterium in addition to *Lactococcus* and *Enterococcus*. Our research results on salinized silage microorganisms were different from those of previous studies. It has been reported that *Lactococcus* and *Lactobacillus* were the main genera of silage after ensiling [33]. Therefore, the role of LAB requires further research to determine whether *Lactobacillus* is suitable for the growth and reproduction of alfalfa in saline soil. Among these LAB, there may be LAB that is halophilic or salt-tolerant lactic acid bacteria.

## Conclusion

The present study illustrated that epiphytic microbiota of forage alfalfa affected the succession of bacterial communities and fermentation quality of the silage. *Enterococcus* and *Lactococcus* dominated the natural fermentation of alfalfa, while *Lactobacillus* and *Pediococcus* constituted the majority of the bacterial community in silages and *Lactococcus* rapidly became the predominant genus in the alfalfa. The use of cellulase enhances the reproduction of *Lactobacillus*. In addition, the fermentation time changes the microbial community of silage fermentation. The results of the study indicate that exogenous epiphytic microbiota of alfalfa under salt stress could be used as a potential bioresource to improve the fermentation quality.

## Supplementary Information

**Additional file 1.** .

**Additional file 2.**

**Additional file 3.** .

**Additional file 4.** .

**Additional file 5.** .

## Data Availability

The data used to support the findings of this study are included within the supplementary information file(s). The nutritional data of alfalfa were measured in the key laboratories of the Ministry of Education and the Ministry of Agriculture and Rural Affairs of China. The remaining alfalfa materials were now stored in laboratory 1043, Biological Science Building, New Campus of Inner Mongolia Agricultural University, Hohhot, Inner Mongolia autonomous Region, China. Contact person, Qiang Lu (Email: 596764747@qq.com.). The microbiological data of the alfalfa was provided by Majorbia Biotechnology Co. (Shanghai, China). Contact person Wei Cao (wei.cao@majorbio.com.). Our datasets are available at NCBI project PRJNA560790.

## Abbreviations

LAB: Lactic acid bacteria
DM: Dry matter
CP: Crude protein
NDF: Neutral detergent fiber
ADF: Acid detergent fiber
WSC: Water-soluble carbohydrates
